# Global perspectives on graduate employability in healthcare services management: insights from Iran

**DOI:** 10.1186/s12913-026-14903-6

**Published:** 2026-06-05

**Authors:** SeyedPouria Hedayati, Soudabeh Hamedi-Shahraki, Fatemeh Mohabati

**Affiliations:** 1 Department of Healthcare Services Management, School of Health, Zabol University of Medical Sciences, Zabol, Iran; 2 Department of Public Health, School of Health, Zabol University of Medical Sciences, Zabol, Iran

**Keywords:** Health services management, Graduate employability, Job market, Mixed-methods research

## Abstract

**Background:**

Healthcare Services Management (HSM) graduates are essential for enhancing healthcare system efficiency but face global employment challenges, including skill mismatches, limited job opportunities, and gender disparities. These barriers hinder their ability to meet workforce demands in diverse healthcare settings. This study investigates these challenges in Iran to inform global HSM education reforms.

**Methods:**

A convergent parallel mixed-methods study at Zabol University surveyed 132 HSM graduates (2010–2022) using the validated “Job Search and Related Factors” questionnaire to quantify employment predictors, such as soft, hard, and academic skills. Concurrently, semi-structured interviews with 37 graduates explored job market experiences, focusing on barriers and facilitators. Quantitative data were analyzed using logistic regression to identify significant predictors, while qualitative data underwent thematic analysis to uncover key themes.

**Results:**

Quantitatively, the employment rate was 63.6%, with Ph.D. holders achieving 85.7%. Soft skills (OR = 1.06, 95% CI [1.02–1.11], *p* = 0.007) and hard skills (OR = 1.14, 95% CI [1.01–1.29], *p* = 0.039) significantly predicted employment, unlike academic skills (*p* = 0.51). Qualitatively, graduates emphasized reliance on social networks (e.g., family connections, veteran quotas), a persistent theory-practice gap in education, and regional isolation in a remote city in southeast Iran limiting opportunities. Women faced cultural barriers to leadership roles despite comparable employment rates.

**Conclusions:**

Addressing skill gaps through practical training, fostering industry partnerships, and promoting gender equity are critical for enhancing HSM graduate employability. These findings offer globally relevant insights for reforming HSM education in less-developed regions, bridging the theory-practice divide.

**Supplementary Information:**

The online version contains supplementary material available at 10.1186/s12913-026-14903-6.

## Background

Healthcare Services Management (HSM) graduates are pivotal in enhancing healthcare system efficiency by integrating clinical and administrative expertise to improve patient outcomes and reduce costs [1, 2]. However, they face global employment challenges, including skill mismatches, limited job opportunities, and gender disparities, which hinder their transition from education to the workforce [3–5]. These issues, while pronounced in certain contexts like Iran, reflect broader trends in healthcare management education and workforce development.

Globally, HSM graduates navigate competitive job markets where academic curricula often fail to meet industry demands [6]. In the U.S., unemployment rates for HSM graduates range from 10 to 15% within six months post-graduation [7], while in developing nations like Tanzania, rates exceed 20% due to resource constraints [6–8]. In Iran, a saturated job market and regional disparities exacerbate these challenges, with unemployment rates reaching 30% in less-developed regions [9]. Key barriers include inadequate practical training, overemphasis on theoretical knowledge, and limited industry partnerships, underscoring the need for curriculum reforms [2, 10].

HSM programs vary by context. In the U.S., curricula prioritize digital competencies and internships [11], while in the UK, leadership and policy analysis are emphasized [12]. In China, programs align with national health priorities [13], and in Tanzania, community health management is critical [8]. Despite these differences, all programs struggle to bridge the theory-practice gap, with employers valuing soft skills (e.g., communication, teamwork) [14], hard skills (e.g., data analysis) [15], and academic skills (e.g., policy knowledge) [16]. These variations highlight the need for globally relevant, context-specific training [17].

Gender disparities further complicate employability. Women, comprising 71% of the global healthcare workforce [18], face barriers to leadership roles, holding only 25% of senior positions [19]. In Iran, cultural norms and workplace biases limit women’s advancement in remote regions, despite high enrollment in HSM programs [20–22]. These trends align with global patterns, where mentorship and policy reforms are needed to promote equity [23, 24].

In Iran, research on HSM graduate employability is limited, particularly in aligning education with market needs in deprived regions [25]. This study addresses this gap by examining factors—skills, social networks, gender, and regional conditions—affecting HSM graduate employability at Zabol University of Medical Sciences (ZBMU). Grounded in human capital theory [26] and employability frameworks [27], it uses a convergent parallel mixed-methods approach to quantify employment predictors (quantitative) and explore job market experiences (qualitative). Findings from this marginalized region aim to inform universal strategies for enhancing HSM education, emphasizing practical training and industry collaboration to meet global healthcare demands [28, 29].

## Methods

### Study design

This study employed a convergent parallel mixed-methods design to investigate the employability of Healthcare Services Management (HSM) graduates at Zabol University of Medical Sciences (ZBMU), Iran. The quantitative and qualitative components pursued distinct but complementary objectives—quantifying employment predictors and exploring lived job market experiences, respectively—integrated to provide a comprehensive understanding of employment barriers and facilitators. Quantitative data identified predictors (soft, hard, and academic skills) using logistic regression, while qualitative data explored perceptions of job market challenges through thematic analysis of semi-structured interviews. Data were collected concurrently, analyzed separately, and integrated during interpretation, aligning with mixed-methods approaches in health management research.

### Quantitative phase

- **Purpose**: To measure employment rates and identify skills (soft, hard, academic) predicting job acquisition.
- **Design**: Cross-sectional survey.
- **Participants and Sampling**: A sample of 132 HSM graduates (2010–2022) was selected via stratified random sampling (strata: gender, graduation year) from the ZBMU alumni database. A power analysis (80% power, α = 0.05) justified the sample size for detecting moderate effects. A 92% response rate was achieved through email and online outreach.
- **Data Collection**: The validated “Job Search and Related Factors” questionnaire [30] comprised 30 items: demographics (10 items: gender, age, GPA, education level, employment status) and skills (20 items: soft skills like communication, hard skills like software proficiency, academic skills like theoretical knowledge) assessed via 5-point Likert scales and categorical questions. Surveys were distributed online using Google Forms.
- **Data Analysis**: Descriptive statistics summarized demographics. Independent t-tests compared skill scores between employed and unemployed graduates, with normality confirmed via Shapiro-Wilk tests. Logistic regression modeled the binary outcome (employed/unemployed) to assess skill impacts, controlling for confounders (VIF < 2). SPSS v26 was used.

### Qualitative phase

- **Purpose**: To explore graduates’ perceptions of job market barriers and facilitators.
- **Design**: Descriptive phenomenological approach to capture lived experiences.
- **Participants and Sampling**: Thirty-seven graduates were purposively selected from the 132 survey respondents to ensure diversity in gender, employment status, and education level, aligning with the convergent parallel design. Participants were contacted via email or phone.
- **Data Collection**: A semi-structured interview guide, developed through literature review and expert consultation, included six open-ended questions on employment status, factors affecting employment, university education impact, unemployment reasons, social networks, and employability solutions. The guide was validated (CVI = 0.88) and pilot-tested with five graduates. Interviews (30–45 min) were conducted via telephone, recorded with consent, and transcribed verbatim until data saturation was reached.
- **Data Analysis**: Thematic analysis followed Braun and Clarke’s six-step framework [31] using MAXQDA. Two coders achieved high inter-coder reliability (Cohen’s Kappa = 0.85). Trustworthiness was ensured through member checking, peer debriefing, and audit trails. Key themes included “Theory-Practice Gap” and “Social Networks.”

## Results

The study integrated quantitative and qualitative data to identify factors influencing Healthcare Services Management (HSM) graduate employability in a remote region in southeast Iran, offering insights into global HSM education challenges.

### Quantitative results

The quantitative phase measured employment rates and skill predictors among 132 HSM graduates (2010–2022). Table 1 summarizes demographics: 75.0% female, 66.7% married, 73.5% bachelor’s degree holders, mean age 30.3 years, and mean GPA 17.1. The employment rate was 63.6%.

**Table 1:** Demographic characteristics of HSM graduates (*n* = 132)

Characteristic	Value
Gender (Female, %)	75.0%
Marital Status (Married, %)	66.7%
Education Level (Bachelor’s, %)	73.5%
Mean Age (years)	30.3 ± 4.2
Mean GPA	17.1 ± 1.8

Table 2 presents employment status by demographics, showing no significant differences by gender (*p* = 0.598), marital status (*p* = 0.697), or education level (*p* = 0.487).

**Table 2:** Employment status by demographics

Variable	Employed (%)	Unemployed (%)	*p*-value
Gender (Male vs. Female)	69.7 vs. 64.6	30.3 vs. 35.4	0.598
Marital Status (Married vs. Single/Divorced)	67.0 vs. 63.6	33.0 vs. 36.4	0.697
Education (Ph.D. vs. Master’s vs. Bachelor’s)	85.7 vs. 67.9 vs. 63.9	14.3 vs. 32.1 vs. 36.1	0.487

Table 3 compares skill scores, with employed graduates scoring higher in soft skills (*p* = 0.009, Cohen’s d = 0.47) and hard skills (*p* = 0.044, Cohen’s d = 0.39), but not academic skills (*p* = 0.486). Logistic regression (Hosmer-Lemeshow test, *p* = 0.72; pseudo-R² = 0.19) identified soft skills (OR = 1.06, 95% CI [1.02–1.11], *p* = 0.007) and hard skills (OR = 1.14, 95% CI [1.01–1.29], *p* = 0.039) as significant predictors of employment, unlike academic skills (OR = 1.03, 95% CI [0.94–1.13], *p* = 0.51).

**Table 3:** Skill scores by employment status

Skill Type	Employed (*n* = 87)	Unemployed (*n* = 45)	*p*-value	Cohen’s d
Soft Skills	62.6 ± 9.7	57.3 ± 12.8	0.009	0.47
Hard Skills	11.3 ± 2.6	10.3 ± 2.5	0.044	0.39
Academic Skills	15.2 ± 3.1	14.8 ± 3.3	0.486	0.12

### Qualitative results

The qualitative phase explored job market experiences of 37 HSM graduates, identifying seven key themes related to employment challenges and opportunities. These themes, summarized in Table 4, include: (1) impact of university education, reflecting limited practical training; (2) social networks, highlighting reliance on family and professional connections; (3) gender barriers, indicating disparities in leadership roles; (4) regional isolation, restricting job opportunities in a remote region in southeast Iran; (5) unrelated jobs, showing employment outside the field; (6) suggested solutions, proposing enhanced training and industry partnerships; and (7) employment barriers, noting job scarcity and exclusion from advertisements. Graduates frequently described a disconnect between theoretical education and job market needs, dependence on social networks for job acquisition, and cultural barriers limiting women’s access to leadership positions. Regional isolation further constrained opportunities, pushing some graduates toward unrelated jobs, while proposed solutions focused on improving practical skills and university-industry collaboration. These findings are detailed in Table 4 with representative quotes.

**Table 4:** Summary of qualitative findings

Theme	Sub-Theme	Quote	Participant
Impact of University Education	Theory-Practice Gap	“Theories were irrelevant; we needed practical skills.”	Graduate 1 (Unemployed)
Impact of University Education	Practicum Limitations	“Our practicum was limited to one hospital, missing broader exposure.”	Graduate 12 (Unemployed)
Social Networks	Family Connections	“My father’s connections secured my job.”	Graduate 2 (Employed, Administrator)
Social Networks	Professional Networking	“Internship relationships led to my job offer.”	Graduate 16 (Employed)
Gender Barriers	Leadership Exclusion	“As a woman, I’m pushed to clerical roles, not leadership.”	Graduate 3 (Employed, Support Role)
Regional Isolation	Geographic Limitations	“Isolation limits jobs in this remote region.”	Graduate 4 (Unemployed)
Unrelated Jobs	Non-Field Employment	“I settled for a job unrelated to my degree.”	Graduate 9 (Employed, Non-HSM)
Suggested Solutions	Industry Partnerships	“University-hospital partnerships would create more opportunities.”	Graduate 18 (Unemployed)

## Discussion

This study investigated the employability of Healthcare Services Management (HSM) graduates in a remote region in southeast Iran, integrating quantitative and qualitative findings to offer insights into global HSM education challenges. The convergent parallel mixed-methods design enabled a comprehensive understanding of employment predictors and lived job market experiences, aligning with mixed-methods approaches in health management research. The findings highlight skill gaps, social networks, gender barriers, and regional constraints as key factors influencing employability, with implications for curriculum reform and policy development worldwide.

Quantitatively, the study identified soft skills (OR = 1.06, 95% CI [1.02–1.11], *p* = 0.007) and hard skills (OR = 1.14, 95% CI [1.01–1.29], *p* = 0.039) as significant predictors of employment, while academic skills were non-significant (*p* = 0.51). These results align with global studies emphasizing the importance of interpersonal and technical competencies in healthcare management [32, 33]. For instance, a U.S.-based study found that communication and teamwork skills were critical for HSM graduate employability [34], while a Tanzanian study highlighted technical skills like data analysis as key differentiators [6]. The 63.6% employment rate in this study, though comparable to regional rates in less-developed areas [35], underscores the need for targeted skill development to bridge the gap between education and job market demands.

Qualitatively, seven themes emerged, including a prominent “Theory-Practice Gap,” where graduates reported limited practical training, echoing findings from China and the UK [35, 36]. The reliance on social networks for job acquisition, another key theme, mirrors studies in low-resource settings where personal connections often compensate for structural barriers [7, 37, 38]. Gender barriers, particularly the relegation of women to support roles (e.g., clerical or operational positions), align with global evidence of leadership disparities in healthcare [39, 40]. For example, a study in India noted that cultural norms restrict women’s access to managerial roles [41], a pattern evident in this study’s remote region. Regional isolation further exacerbated employment challenges, consistent with research on geographic disparities in healthcare workforce distribution [42].

### Global implications

These findings position the study as a case study for global HSM education reform. The emphasis on soft and hard skills suggests that curricula should prioritize experiential learning, such as internships and simulations, to address the theory-practice gap [43]. University-industry partnerships, as suggested by graduates, could facilitate job placements and align training with market needs, a model successful in countries like Australia [44]. Addressing gender barriers requires policies promoting equitable leadership opportunities, such as mentorship programs for women, which have proven effective in Western contexts [45]. While regional isolation poses unique challenges, virtual training and remote job platforms could mitigate geographic constraints, as demonstrated in rural healthcare initiatives [46].

### Limitations

Several limitations should be noted. As qualitative participants were a subset of survey respondents, perspectives of graduates who did not complete the survey may be underrepresented, though purposive sampling ensured diversity. The study’s focus on a remote region in southeast Iran limits generalizability, but comparisons with global literature enhance its broader relevance. The absence of employer perspectives restricts insights into hiring criteria, a gap future research could address. Time constraints prevented longitudinal follow-up, which could reveal long-term employment trends.

## Conclusion

This study underscores the need for HSM programs to align with job market demands through practical training, equitable policies, and strategic partnerships. By addressing skill gaps, gender disparities, and regional barriers, HSM education can better prepare graduates for diverse healthcare settings worldwide, contributing to a more competent and inclusive workforce.

## Supplementary Information

Below is the link to the electronic supplementary material.

Supplementary Material 1

## Data Availability

The dataset used and analyzed during the current study is available from the corresponding author upon reasonable request.
