# Supplementary material for: Global perspectives on graduate employability in healthcare services management: insights from Iran
Source: BMC Health Serv Res. 2026 Jun 5;26:1106. doi: 10.1186/s12913-026-14903-6 (PMC13471670; doi:10.1186/s12913-026-14903-6)
Supplement: Supplementary file 1 [file 12913_2026_14903_MOESM1_ESM.docx]

*(Adapted from Nozari et al. (2022) and localized for graduates of Healthcare Services Management at Zabol University of Medical Sciences)*

**Introduction:**
This questionnaire assesses the employability of Healthcare Services Management graduates from Zabol University of Medical Sciences, focusing on demographic factors and skills relevant to the Iranian healthcare system. It includes two primary sections: Demographic Information and Skills Evaluation.

**Section 1: Demographic Information**

1. Gender: [1] Female [2] Male
2. Marital Status: [ ] Single [ ] Married [ ] Divorced
3. Highest Level of Graduation:
   [1] Bachelor’s [2] Master’s [3] PhD
4. Employment Status:
   [1] Employed [2] Unemployed
5. Age: ____________________ years
6. GPA: ____________________

**Section 2: Skills Evaluation**

7. To what extent is your current job related to your Healthcare Services Management degree?
   [1] Very Little [2] Little [3] Moderate [4] Much [5] Very Much
8. Did you acquire the skills needed for your job during your studies at Zabol University?
   [1] Yes [2] No
9. How important are the following soft skills in your employment?
   (Scale: 1 = Very Little, 2 = Little, 3 = Moderate, 4 = Much, 5 = Very Much)

| **No.** | **Skill** | **1** | **2** | **3** | **4** | **5** |
| --- | --- | --- | --- | --- | --- | --- |
| 1 | Communication skills |  |  |  |  |  |
| 2 | Teamwork |  |  |  |  |  |
| 3 | Problem-solving skills |  |  |  |  |  |
| 4 | Decision-making skills |  |  |  |  |  |
| 5 | Work ethics |  |  |  |  |  |
| 6 | Leadership skills |  |  |  |  |  |

10. How important are the following hard skills in your employment?
    (Scale: 1 = Very Little, 2 = Little, 3 = Moderate, 4 = Much, 5 = Very Much)

| **No.** | **Skill** | **1** | **2** | **3** | **4** | **5** |
| --- | --- | --- | --- | --- | --- | --- |
| 1 | Computer proficiency |  |  |  |  |  |
| 2 | Familiarity with healthcare technologies |  |  |  |  |  |
| 3 | Proficiency in healthcare management software |  |  |  |  |  |

11. How much have the following academic skills impacted your employment?
    (Scale: 1 = Very Little, 2 = Little, 3 = Moderate, 4 = Much, 5 = Very Much)

| **No.** | **Skill** | **1** | **2** | **3** | **4** | **5** |
| --- | --- | --- | --- | --- | --- | --- |
| 1 | Proficiency in software related to healthcare management |  |  |  |  |  |
| 2 | Theoretical knowledge of healthcare management |  |  |  |  |  |
| 3 | GPA during studies |  |  |  |  |  |
